# OPSI threat in hematological patients

**Published:** 2013-05-06

**Authors:** B Serio, L Pezzullo, V Giudice, R Fontana, S Annunziata, I Ferrara, R Rosamilio, C De Luca, M Rocco, N Montuori, C Selleri

**Affiliations:** 1 Hematology and Hematopoietic Stem Cell Transplant Center, Department of Medicine and Surgery, University of Salerno, Salerno, Italy;; 2 Department of Translational Medical Sciences, Federico II University of Napoli, Napoli, Italy.

**Keywords:** Overwhelming postsplenectomy infection (OPSI) syndrome, splenectomy, hematological disorders

## Abstract

Overwhelming post-splenectomy infection (OPSI) is a rare medical emergency, mainly caused by encapsulated bacteria, shortly progressing from a mild flu-like syndrome to a fulminant, potentially fatal, sepsis. The risk of OPSI is higher in children and in patients with underlying benign or malignant hematological disorders.

We retrospectively assessed OPSI magnitude in a high risk cohort of 162 adult splenectomized patients with malignant (19%) and non malignant (81%) hematological diseases, over a 25-year period: 59 of them splenectomized after immunization against encapsulated bacteria, and 103, splenectomized in the previous 12-year study, receiving only life-long oral penicillin prophylaxis. The influence of splenectomy on the immune system, as well as the incidence, diagnosis, risk factors, preventive measures and management of OPSI are also outlined.

OPSI occurred in 7 patients (4%) with a median age of 37 years at time interval from splenectomy ranging from 10 days to 12 years. All OPSIs occurred in non immunized patients, except one fatal *Staphylococcus aureus* -mediated OPSI in a patient adequately immunized before splenectomy.

Our analysis further provides evidence that OPSI is a lifelong risk and that current immune prophylaxis significantly decreases OPSI development.

Improvement in patients’ education about long-term risk of OPSI and increased physician awareness to face a potentially lethal medical emergency, according to the current surviving sepsis guidelines, represent mandatory strategies for preventing and managing OPSI appropriately.

## I. INTRODUCTION

**Figure 1.**
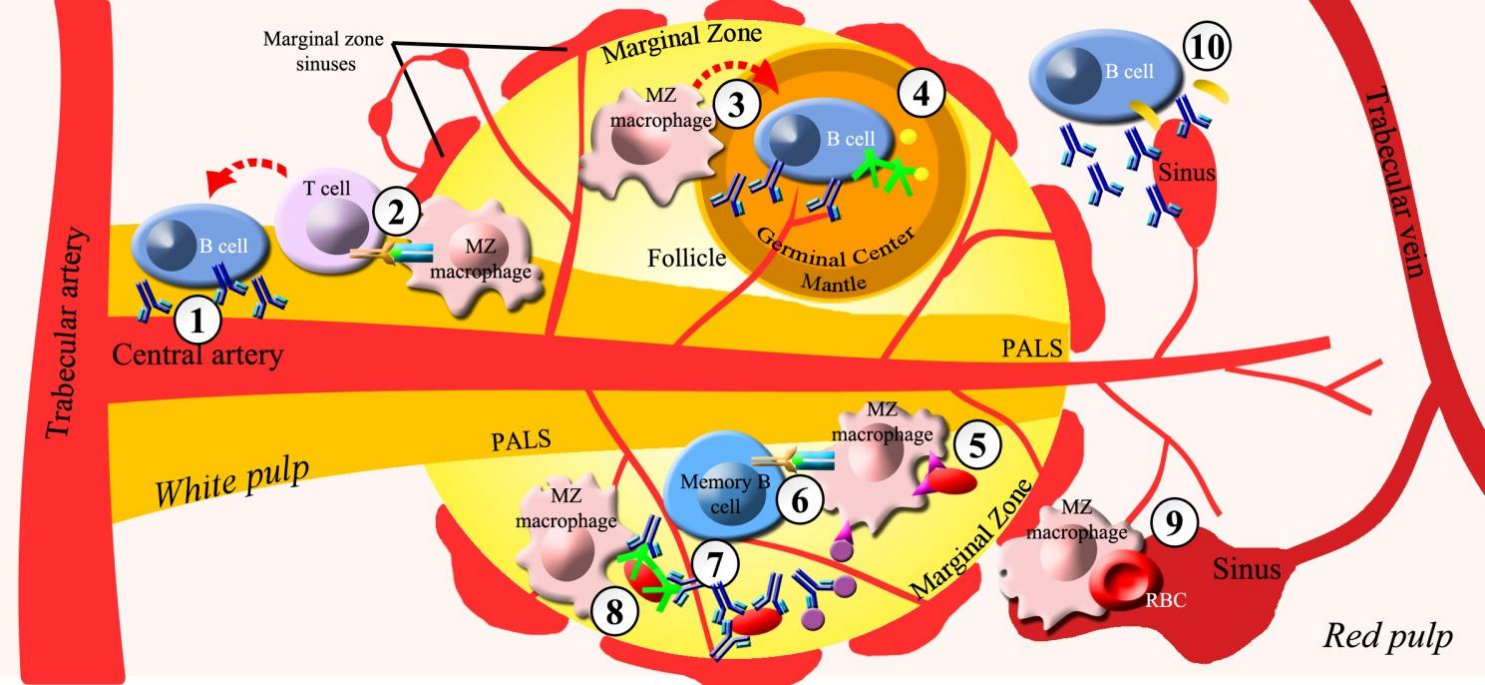
**
Antimicrobial splenic functions.** Compartments of white pulp involved in protective antimicrobial functions: a) periarteriolar lymphoid sheats (PALS) through ① secretion of T-dependent immunoglobulins (Ig) and ② antigen presentation to T-Lymphocyte by marginal zone (MZ) macrophages; b) follicles through ③ MZ macrophage-mediated activation of B lymphocytes and plasmacells and ④ production of antibodies, complement and opsonins; c) marginal zone through ⑤ opsonin-independent phagocytosis mediated by MZ macrophages, ⑥ presentation of particulate antigens by MZ macrophages to CD27+IgM+ B cells, ⑦ rapid release of antibodies by CD27+IgM+ memory B cells, ⑧ opsonin-dependent phagocytosis by MZ macrophages. Red pulp exerts anti-microbial protective functions mainly through ⑨ blood-borne pathogens filtering, culling and pitting of red blood cells (RBC) and ⑩ rapid GM-CSF release *via* innate response activator (IRA) and antibodies by B cells and plasmacells. **
Symbols:** Ig = 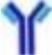; T-cell receptor/Major hystocompatibility complex binding, 
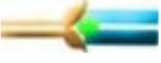
; complement, 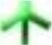;opsonin, 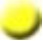; pathogen-recognition-receptors (PRRs), 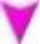; particulate antigen, 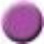; GM-CSF (Granulocyte-macrophage colony-stimulating factor), 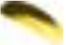.

The spleen, through its two main compartments called red and white pulp, is the largest secondary lymphoid organ dedicated to the blood filtering of old or damaged blood cells and foreign pathogens as well as to the immune surveillance and response to infections. Although, rapid removal of infectious agents from circulation by red pulp of the spleen is the prerequisite for successful control of infection, the key events responsible for initiating host defence against pathogens are developed in sub-compartments of white pulp distributed along central arterioles, including the periarteriolar lymphoid sheaths (PALS), follicles, and marginal zone ( Figure 1 , Table 1 ) [ 1 – 6 ] .

The splenic marginal zone (MZ) is the boundary between the red and white pulp where the terminal arterioles open into sinuses and the blood flow is slowed down, favouring early trapping of blood-borne bacteria, viruses, parasites, and other soluble and particulate antigens by dendritic cells, metallophilic macrophages and MZ macrophages [ 7 ] . Splenic MZ macrophages are highly phagocytic cells and are responsible for rapid clearance of blood-borne T-independent antigens and debris, by virtue of expressing an array of receptors promoting efficient antigen uptake detecting invading pathogens either directly or through intermediate opsonins [ 7 – 10 ] . Pathogen-recognition-receptors (PRRs), mediating opsonin-independent phagocytosis by splenic MZ macrophages, include Toll-like receptors, C-type lectins, type I and II scavenger receptors, macrophage receptors with collagenous structure, CD36, the mannose receptor and others [ 4 , 7 , 11 , 12 ] . Deficit of these PRRs increases susceptibility to a variety of viral, bacterial and mould infections [ 13 – 15 ] . Splenic MZ macrophages are also involved in opsonin-dependent phagocytosis, via natural antibodies and components of the complement system, which plays a critical role in the early control of bacterial and viral infections [ 16 , 17 ] . In addition, splenic MZ macrophages cooperate to deliver antigen presentation to T-cell areas (necessary for triggering protective T cell responses) [ 7 , 9 ] such as PALS and a thin layer surrounding B-cell follicles, formed by an outer mantle zone containing dominantly proliferating small B lymphocytes and a central germinal center where B-cell selection occurs [ 2 , 4 , 6 ] .

Several lines of evidence link a reduced antigen trapping by the splenic MZ macrophages to an impairment of early control of infection enhancing spread of the pathogens into circulation and organs [ 18 ] . Moreover, the human splenic MZ is a unique B-cell compartment containing a large population of immunoglobulin (Ig) M+, IgD+, CD27+ (IgM ^+^ IgD ^+^ CD27 ^+^ ) “memory” B cells, carrying a mutated immunoglobulin receptor, distinct from the classical germinal center–derived memory B cells, which can be rapidly mobilized and activated to secrete Ig in response to blood-borne T-independent antigens [ 19 – 22 ] . However, MZ B cells have also been shown to contribute to the generation of T cell-dependent antibody responses ( Figure 1 , Table 1 ) [ 9 , 23 ] . The result of this complex cross talk between white and red pulp as well as B and T cell areas of white pulp, linking innate and adaptive immune response, is thought to predominantly contribute to the sepsis-protective effect of the spleen [ 9 , 24 ] .

Overwhelming post-splenectomy infection (OPSI) is a rare fulminant sepsis with a mortality up to 70% [ 25 – 27 ] . An underlying hematological disease and conditions eliciting immunosuppression are associated with higher OPSI rate and mortality, with thalassemic and drepanocytic patients having the highest rates of infections (8.2% and 7.3%) and mortality (5.1% and 4.8%), respectively [ 16 , 28 ] . OPSI and mortality rate in splenectomized patients for autoimmune thrombocytopenia are similar to that for trauma (about 2.3% and 1.2%,) and lower than that for Hodgkin’s disease (4.1% and 1.9%), and spherocytosis (3.1% and 1.3%) [ 28 ] .

Apart from splenectomized subjects, higher rate of infections have been also reported in diseases associated with splenic atrophy or hyposplenism, such as celiac disease, Crohn’s disease, sickle cell anemia, spherocytosis, chronic graft *versus* host disease, systemic lupus erythematosus, HIV infection, amyloidosis and several other disorders ( Table 2 ) [ 1 , 6 ] . The cumulative risk for asplenic and hypospenic patients to develop an OPSI is estimated to be about 1 per 500 patients per year [ 26 , 29 ] .

Although the risk of infections declines with the time elapsed following splenectomy, with up to 50% of cases occurring within 2 years after surgery, 30–40% of OPSI have been documented > 5 years and individual cases even more than 40 years post-splenectomy, indicating a long-lasting infection risk in these patients [ 30 ] .

Most OPSI are caused by encapsulated bacteria such as *Streptococcus pneumoniae* (involved in more than 50% of cases), *Haemophilus influenzae* , *Neisseria meningitidis* , but also by intraerythrocytic parasites or bacteria mainly removed by the spleen, such as *Babesia* , *Ehrlichia* and *Plasmodia* [ 19 , 26 , 29 , 31 – 33 ] . *Enterococcus* , *Bacteroides* , *Salmonella* and *Bartonella* species have occasionnally been reported responsible for OPSI [ 31 ] . Recently, OPSI cases due to the Gram negative bacterium *Capnocytophaga canimorsus* , transmitted through dog, cat or other animal bites have also been reported [ 29 , 31 , 34 ] .

Early stage of OPSI is usually characterized by aspecific symptoms similar to those of a flu-like syndrome quickly progressing to symptoms of a bacteremic septic shock, including hypotension, acidosis, electrolytic disorder, hypoglycemia, oliguria, respiratory distress, cardio-vascular collapse, convulsions, disseminated intravascular coagulopathy (DIC) and multi-systemic organ failure. Coma and death can occur within 24 to 72 hours of illness onset [ 19 , 25 ] . Children more frequently than adults have concomitant pneumonia and meningitis [ 35 ] .

**Figure 2.**
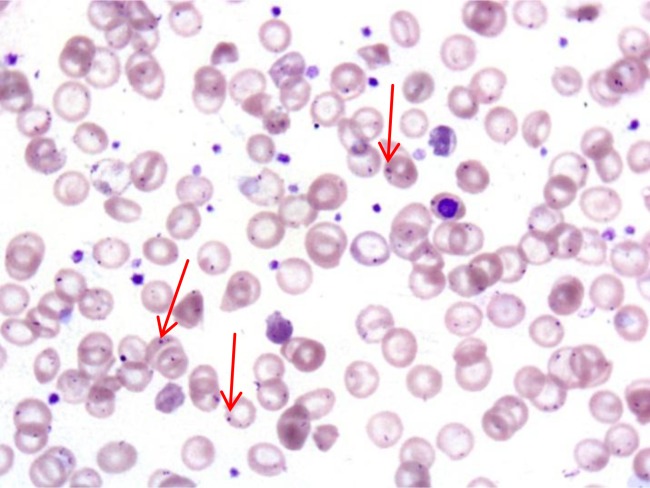
**
Howell-Jolly bodies.** Howell-Jolly bodies (red arrows) in circulating red blood cells of a patient with OPSI.

Supporting laboratory evidences of OPSI, apart from those specifically related to the bacteremic septic shock, DIC and multi-system organ failure, include the presence of Howell-Jolly bodies in erythrocytes [ 6 , 19 , 34 , 36 ] ( Figure 2 ), and of pitted erythrocytes [ 5 , 19 , 37 ] .

Other investigations useful for splenic function assessment, such as scintigraphic and immunological parameters, detailed in Table 3 , are limited by their technical difficulties in critically ill patients, such as those suspected of having a OPSI [ 6 , 19 , 37 – 38 ] .

## II. METHODOLOGY

### Subjects

We have conducted a retrospective evaluation of adult patients who were splenectomized (n=162) over a 25-year period for hematological diseases ( Table 4 ). A few cases of splenectomy in children are reported in our survey (11 cases < 15 years), coming to our attention months or years after surgery. Informed consent was obtained from all patients in accordance with institutional guidelines and the study design was made in accordance with the Helsinki II Declaration [ 39 ] .

The age at splenectomy ranged between 11 and 60 years (mean±SD, 37±22 years) and their post-splenectomy follow-up lasted from 1 to 300 months (mean±SD, 150±12 months). Primary diseases were immune thrombocytopenia (ITP; n=86), Hodgkin and non Hodgkin’s lymphoma (HD/NHL; n=22), warm antibody autoimmune hemolytic anemia (wAIHA; n=14), hereditary spherocytosis (HS; n=10), Cooley’s disease (n=10), congenital non-spherocytic hemolytic anemias (CNSHAs; n=5), idiopathic myelofibrosis (IMF; n=5), hairy cell leukemia (HCL; n=4), β-thalassemia intermedia (n=4) and sickle cell anemia (n=2). In adult ITP, most of splenectomy (76% of cases) was performed on adults who did not respond to two courses of glucocorticoid treatment or who continued to require high–dose steroids to achieve a safe platelet count. In the remaining and more recent ITP cases, although the correct positioning of rituximab or thrombopoietin receptor agonists for the management of chronic ITP before splenectomy is not yet clear, surgery was performed after the failure of such therapies for refusal of patients to undergo surgery as second or third-line therapy [ 30 , 40 – 43 ] . In wAIHA, splenectomy was performed long time after the beginning of the disease (at least 1 year) in patients with refractory or recurrent disease after immunosuppressive therapies including steroids, azathioprine, cyclophosphamide, rituximab, and requiring high maintenance steroid doses to achieve an acceptable hemoglobin value [ 30 , 45 – 47 ] . In these wAIHA, splenectomy was performed only after measurement of ^51^ Cr-red blood cell sequestration in the spleen [ 48 ] . The majority of cases of splenectomy for thalassemia and hemoglobinopathies, including Cooley’s disease, intermediate thalassemia and sickle cell anemia, refer to patients who already underwent surgery in pediatric age for progressive increase in transfusion requirement and difficulty to control iron overload [ 32 , 49 ] . In hereditary spherocytosis and other CNSHAs, including pyruvate kinase deficiency, splenectomy was performed when patients had moderate/severe anemia [ 50 – 51 ] . In CNSHAs, like in wAIHA, also splenectomy was performed only after measurement of ^51^ Cr-red blood cell sequestration in the spleen [ 48 ] . In 22 patients with HD and NHL, splenectomy was mainly diagnostic, in few HD and NHL cases also therapeutic [ 52 ] . Four patients with HCL were splenectomized in pre-interferon and cladribrine era when they suffered from moderate/severe hypersplenism-related pancytopenia [ 53 ] . Splenectomy was performed in 5 selected IMF patients showing heavily symptomatic splenomegaly refractory to drug treatment, and transfusion-dependent anemia [ 54 ] ( Table 4 ).

Open surgery was conducted in 105 patients, while 57 patients underwent laparoscopic splenectomy.

### Antibiotic prophylaxis and immunization

Prophylactic oral penicillin was prescribed to all splenectomized patients for the first two years after splenectomy or up to age 16 [ 6 , 19 , 55 – 58 ] .

Before or within 2 weeks from surgery, when specific vaccines were commercially available, immunization against *Streptococcus pneumoniae* , *Haemophilus influenzae* type b (Hib) and *Neisseria meningitidis* were carried out using the 23-valent pneumococcal polysaccharide vaccine (23vPVV), Hib protein–polysaccharide conjugate vaccine and 4-valent meningococcal polysaccharide vaccine (4vMenPV), respectively [ 6 , 19 , 55 – 57 , 59 – 61 ] .

### Statistical analysis

All data were collected from a computerized database and chart reviews were analysed using GraphPad Prism version 5. A P value ≤.01 was considered to be statistically significant.

## III. RESULTS

### Patients characteristics

A retrospective observational study evaluating OPSI development rate was performed in 162 adult patients which underwent splenectomy for malignant (19%) and non malignant (81%) hematological diseases over a 25-year period. Most of the patients (n=99, 61%) in our survey underwent splenectomy within the first 12 years of the study. Forty-seven percent of splenectomized patients had a higher risk of developing OPSI, since they were splenectomized for hematological disorders associated with severe impaired immunity such as thalassemia or sickle cell anemia (10%), hereditary spherocytosis or nonspherocytic anemia (9%), autoimmune hemolytic anemia (7%), malignant lymphoproliferative and myeloproliferative diseases (19%) ( Table 4 ).

It has been reported that the infection risk and death rates for ITP patients is similar to those who underwent splenectomy for trauma [ 41 ] . However, almost all our ITP patients were heavily immunosuppressed, since they underwent surgery only after refractoriness to at least two-three courses of immunosuppressive therapy with at least two different drugs or who continued to require long-lasting high-dose steroids to achieve safe platelet counts.

### OPSI prophylaxis

All splenectomized patients received oral penicillin antibiotic prophylaxis for the first two years after splenectomy or up to age 16. In addition, life-long prophylactic oral penicillin was offered to all immunosuppresed patients, but not all of these patients have taken it either due to intolerance or refusal to perform it continuously.

Vaccine prophylaxis against *Streptococcus pneumoniae* was performed in 59 (36%) patients (ITP, n=35; HD/NHL, n=6; wAIHA n=5; HS, n=5; Cooley’s disease, n=3; CNSHA, n=1; IMF, n=2; Thalassemia intermedia, n=2), associated with simultaneous immunization against Hib and *Neisseria meningitidis* in 42 patients (26%) (ITP, n=30; wAIHA n=2; HD, n=2; HS, n=5; Cooley’s disease, n=3) ( Table 4 ).

### OPSI

OPSI occurred in 7 patients (4%) with a median age of 37±22 years and an age range from 16 to 60 years ( Table 5 ). All patients had high risk disease for OPSI including 2 cases with lymphoma, 2 with thalassemia intermedia, 2 with Cooley’s disease, and 1 with long-lasting warm antibody auto-immune hemolysis. The interval from splenectomy to OPSI varied from 10 days to 12 years with 3 OPSI (43%) occurring shortly post-splenectomy (from 10 to 25 days) and 4 OPSI (57%) >5 years post-splenectomy (from 5 to 12 years, respectively) ( Table 5 ).

In all OPSI, the onset of symptoms was mild, like a flu-like syndrome, followed by high fever (even without fever in a case of wAIHA), and by septic shock and DIC leading within 2–5 days to death in 6 out of 7 cases (85%) from an apparent good health. Only a 17 years-old female with Cooley’s disease survived from a group A *Streptococcus* -mediated OPSI developing 7 years post-splenectomy. She recovered following immediate transfer to an intensive care unit where she was treated with prolonged ceftriaxone and vancomycin therapy combined with aggressive management of shock and DIC; currently, she is well 5 years after HLA-matched sibling bone marrow transplantation.

All OPSI occurred in non immunized patients except a OPSI case with a 10-year history of wAIHA, who carried out a pre-splenectomy vaccination program with both anti-pneumococcal and anti-meningococcal vaccines, as well as with anti-Hib vaccine. Apart from this last OPSI, where a *Staphylococcus aureus* bacteremia was detected and the above mentioned group A *Streptococcus* –mediated OPSI, in all other OPSI cases (71%) a pneumococcal bacteriemia was documented.

### Thromboembolic complications

Patients undergoing splenectomy for hematologic disorders have an increased risk of vascular complications [ 30 , 44 ] . In our survey, we have registered 2 fatal vascular complications, both after open splenectomy: the first one in a 41 year old ITP female with delayed signs of mesenteric thrombosis and the second one in a 50 year old IMF male with signs of pulmonary hypertension as a result of in situ thrombosis. A 49 year old male, with acute pulmunary thromboembolism, requiring thrombolytic therapy and systemic anticoagulation during 59 days of hospitalization, followed by oral anticoagulation for 6 months, is currently well 5 years post-splenectomy ( Table 5 ).

## IV. DISCUSSION

Splenectomy provides a high rate of complete response for patients with immune thrombocytopenia [ 41 – 42 ] and can ameliorate the underlying anemia in a variety of other benign hematological disorders including hemolytic anemia, hereditary spherocytic and non spherocytic hemolytic anemias, Cooley’ diseases, thalassemia intermedia and sickle cell anemia [ 30 , 45 – 51 ] . In addition, splenectomy still remains an useful approach for the diagnosis and for relieve symptoms of hypersplenism and splenomegaly in several malignant hematological diseases such as lymphomas and chronic myeloproliferative syndromes ( Table 2 ) [ 52 – 54 ] .

Life-threatening infections and thromboembolic events represent the major short- and long-term postoperative complications after splenectomy [ 30 , 44 ] ; additionally, severe infections may affect clinical course of several functional hyposplenisms ( Table 2 ) [ 1 , 6 ] .

OPSI, a rare clinical infectious syndrome, affecting about 3% of splenectomized patients, is a serious medical emergency shortly progressing to a fulminant sepsis mainly by encapsulated bacteria [ 19 , 26 , 31 – 33 ] , carrying a high mortality rate (>50%) [ 16 , 26 , 28 – 29 ] .

The major documented risk factors for OPSI development in asplenic patients are younger age, underlying diseases, previous and concomitant immunosuppression and time from splenectomy [ 34 ] .

Splenectomy is usually postponed until the child is 6 year old, due to spleen immaturity in the first 5 years of age, mainly related to low levels of opsonin- and IgM memory B cells responsible for phagocytosis of encapsulated bacteria [ 37 ] . Although in our series, children suffering from hereditary hematological disorders underwent splenectomy when they were more than 5 year-old, we registered in the pre-vaccination era, 3 fatal OPSI despite they had been receiving life-long antibiotic prophylaxis, and careful quantification of Howell-Jolly bodies on peripheral blood films during the whole follow-up [ 36 ] .

Early increase of circulating Howell-Jolly bodies and pitted erythrocytes after splenectomy are historically associated, when their percentage is more than 8%, with significant splenic dysfunction [ 36 – 37 ] . Recently, measurement of circulating IgM ^+^ IgD ^+^ CD27 ^+^ “memory” B cells has also been proposed as an assay to assess asplenic populations at risk for OPSI. However, although IgM memory B cells gradually decrease within 6 months after splenectomy achieving reduced stable levels up to more than 2 years, no association of levels of IgM memory B cells with the underlying indication for splenectomy and infections has been reported [ 20 – 21 ] .

In our cohort of 162 high risk splenectomized patients for malignant (19%) and non malignant (81%) hematological disorders, OPSI occurred in 4% of cases with a median age of 37±22 years.

Previous reports have consistently documented that the highest risk of infections following splenectomy, is within the first two years after surgery. In our series, OPSI occurred early (within 1 month) in 43% of cases and late (over 5 years, 57%) in the remaining, further indicating a long-lasting OPSI risk in these patients ( Table 5 ). In accordance with literature, we also documented that the most frequent etiologic agent responsible for OPSI was the *Streptococcus pneumoniae* (70% of cases) [ 19 , 26 , 31 ] .

Various guidelines for the prevention of sepsis in asplenic patients exist in the literature; these recommendations are based on immunization against *S. pneumoniae, N. meningitidis, H. influenzae* type b, lifelong antibiotic prophylaxis in high risk patients [ 6 , 19 , 55 – 57 , 59 – 61 ] , and education of patients and health professionals to prevents infections [ 6 , 62 – 64 ] .

Since in our survey, 70% of hematological patients underwent splenectomy within the first 12 years of the study, a time when the current polyvalent pneumococcal vaccine was not commercially available, only 35% of them underwent surgery prior to anti-pneumoccoccal vaccination, associated with simultaneous anti-meningococcal and anti-influenza type B immunization in 25% of cases. In our series, OPSI development among patients immunized and those not receiving vaccinations significantly decreased (from 5% to 1.7%, respectively; p<.01). Recently, an increasing number of OPSI cases caused by pathogens not covered by vaccinations or resistant to antibiotic prophylaxis has been reported [ 17 , 19 , 26 , 29 , 31 – 34 ] . In our series, we documented a case of fatal *Staphylococcus aureus* -mediated OPSI in a patient who had undergone a complete pre-splenectomy vaccination program, according to the current guidelines.

Although the introduction of an adequate vaccination program against encapsulated bacteria and systematically oral penicillin prophylaxis have decreased the overall risk of OPSI, its mortality rate remains high, ranging from 50% to 70% within 48 h [ 55 ] .

OPSI, once triggered, is a serious medical emergency requiring immediate therapeutic administration of antimicrobial agents and aggressive care support according to surviving sepsis campaign guidelines for management of severe sepsis and septic shock, even before any diagnostic test result performed upon admission comes back from laboratories [ 25 , 63 ] . Observational evidences have documented that early aggressive antimicrobial therapy combined with intensive care support may decrease the mortality rate for OPSI as low as 10% [ 25 ] .

Thromboembolic complications following splenectomy for hematological diseases represent another short- and long-term life-threatening complication of splenectomy; they occurs in up to 10% of these patients, more frequently among myeloproliferative and hemolytic patients, ranging from portal vein thrombosis to pulmonary embolism [ 30 , 44 ] . In our series, we documented 3 severe thromboembolic events: two of them shortly and one after a prolonged time interval (3 years) after surgery for IMF and ITP, respectively. Recently, Crary et al. [ 64 – 66 ] as well as Di Sabatino et al. [ 6 ] reviewed this issue addressing specific recommendations for post-splenectomy thromboembolism prevention and management.

Increased awareness that splenectomized patients face lifelong risk of OPSI and vascular complications, have led to a more conservative approach, resulting in a significant decrease of splenectomies in the last 15 years for emergency, especially for treatment of benign hematologic disorders [ 44 ] . In our series of adult hematologic patients, only 59 of them underwent splenectomy in the last 12 years.

In addition, various surgical techniques of partial splenectomy, preserving 10–20% of functional splenic tissue, have been recently adopted, but their role on prevention of OPSI and vascular complications is still controversial [ 67 – 69 ] .

## V. CONCLUSION

Splenectomy and hyposplenism are associated with multiple and complex abnormalities of innate and adaptive immune response, including mainly decreased filtering of blood-borne pathogens and T-dependent and T-independent antibody production by IgM ^+^ IgD ^+^ CD27 ^+^ memory B cells against encapsulated bacteria. Lifelong risk for overwhelming sepsis and, to a lesser extent, for thromboembolism remains major complications after splenectomy occurring irrespective of age and time interval after the surgical procedure.

The risk of OPSI is higher in children less than 5 years old, in those who have had immunosuppressive treatments as well as in patients suffering from a benign or malignant hematological disorder.

Currently, the established standard of care for post-splenectomy patients includes immunization with polyvalent pneumococcal vaccine, *Haemophilus influenzae* type b conjugate and meningococcal polysaccharide vaccine before or within 2 weeks from splenectomy, associated with prophylactic penicillin, and patient education to alert physicians when fever occurs.

Despite the above mentioned preventive measures decreased the overall risk of OPSI, it is still associated with an unacceptably high mortality rate.

Concerns about the highest OPSI mortality rate among hematologic patients and the increasing appearance of OPSI mediated by pathogens not covered by current vaccinations led to a more conservative approach through a reassessment of indications and timing of the surgery, given also the expanding arsenal of therapeutics available for treating hematological disorders. Alternative methods for preventing post splenectomy sepsis, such as subtotal splenectomy, need to be confirmed in larger studies.

Despite various limitations, including retrospective analysis, a small number of patients, especially in the group receiving the appropriate vaccination program, our study further provides evidence that OPSI is a lifelong risk and that immune prophylaxis against encapsulated bacteria significantly decreases OPSI development among hematological patients.

Early identification and aggressive emergency treatment of OPSI with antimicrobial agents and intensive care support may positively affect outcomes of this fulminant and fatal disease.

Improvement of patients’ education about their asplenic or hyposplenic status and increased healthcare worker awareness about the potential fulminant progression of infections in splenectomized patients represent mandatory strategies for preventing and managing OPSI appropriately.

## Figures and Tables

**TABLE 1:** PROTECTIVE ANTIMICROBIAL FUNCTIONS OF THE SPLEEN

** Pulp **	** Functions **
** Red **	- Blood-borne pathogens filtering
	- Culling and pitting of red blood cells
	- Rapid GM-CSF release * via * IRA
	- Rapid antibodies release * via * B cells and plasmacells

** White **	*** Periarteriolar lymphoid sheath (PALS): ***
	- Antigen presentation to T-lymphocytes by MZ macrophages
	- T-dependent Ig secretion
	*** Follicles: ***
	- Storage and maturation of B lymphocytes
	- Antibodies release by B lymphocytes
	- Production of complement, opsonins, properdin and tuftsin
	*** Marginal zone: ***
	- Antigen presentation by MZ macrophages to CD27 ^ + ^ IgM ^ + ^ B cells
	- Trapping of pathogens by MZ dendritic cells, metallophilic and MZ macrophages
	- Opsonin-dependent phagocytosis * via * complement and antibodies by MZ macrophages
	- Opsonin-independent phagocytosis * via * PRRs by MZ macrophages
	- Rapid clearance of blood-borne T-independent antigens
	- Rapid release of antibodies by CD27 ^ + ^ IgM ^ + ^ B cells

**Abbreviations.** GM-CSF = granulocyte - macrophages colony - stimulating factor; IRA = innate response activator; MZ = marginal zone; CD = cluster of differentiation; Ig = immunoglobulins; PRRs = pathogen-recognition-receptors.

**TABLE 2:** DISORDERS ASSOCIATED WITH HYPOSPLENISM

** Disorder **	** Disease **
* Congenital *	Congenital asplenia Congenital cyanotic heart disease
* Hematologic *	Sickle cell anemia B-Thalassemia Hereditary spherocytosis Graft * versus * host disease Autoimmune Hemolytic Anemia Immune Thrombocytopenia Mastocytosis
* Oncologic *	Breast cancer Angiosarcoma Splenic hemangiosarcoma Splenic hemangioendothelioma
* Gastrointestinal *	Coeliac disease Ulcerative colitis Crhon’s disease
* Hepatic *	Chronic active hepatitis Liver cirrhosis and portal hypertension
* Autoimmune *	Systemic lupus erythematosus Antiphospholipid syndrome Vasculitis Rheumatoid arthritis Mixed connective tissue disease
* Infectious *	Acquired immunodeficiency syndrome
* Circulatory *	Splenic artery thrombosis Splenic vein thrombosis
* Miscellaneous *	Sarcoidosis Amyloidosis Surgical splenectomy Primary pulmonary hypertension

**TABLE 3:** DIAGNOSTIC TECNIQUES ASSESSING SPLENIC FUNCTION

- Detection of circulating Howell-Jolly bodies on stained blood films - Detection of pitted erythrocytes by phase-interference microscopy - Detection of B cells CD27 ^+^ IgM ^+^ decrease by flow-cytometry - Technetium-99m-labelled sulphur colloidal scintiscan - Clearance of Technetium-99m-labelled or rubidium -81-labelled heat-damaged autologous erythrocytes

**TABLE 4:** UNDERLYING DISEASES OF SPLENECTOMIZED PATIENTS

** Disease **	** Cases (n) **
Immune thrombocytopenia	86
Hodgkin’s and non-Hodgkin’s lymphoma	22
Warm antibody autoimmune hemolytic anemia	14
Hereditary spherocytosis	10
Cooley’disease	10
Congenital non-spherocytic hemolytic anemias	5
Idiopathic myelofibrosis	5
Hairy cell leukemia	4
Thalassemia Intermedia	4
Sickle cell anemia	2
** Total **	162

**TABLE 5:** OVERWHELMING SEPSIS AND THROMBOTIC EVENTS AFTER SPLENECTOMY

** Event **	** Age **	** Disease **	** Timing post surgery **	** Outcome **
OPSI	45	HD	10 days	dead
OPSI	16	Cooley	10 days	dead
OPSI	60	NHL	25 days	dead
OPSI	18	Th. Int.	12 years	dead
OPSI	34	wAIHA	5 years	dead
OPSI	28	Th. Int.	6 years	dead
OPSI	17	Cooley	8 years	alive
Pulmonary thrombosis	50	IMF	3 years	dead
Mesenteric thrombosi	45	ITP	2 months	dead
Pulmonary thrombosis	49	IMF	21 days	alive

**Abbreviations** . OPSI = overwhelming post-splenectomy infection; HD = Hodgkin’s disease; NHL = non Hodgkin’s lymphoma; Th.Int. = thalassemia intermedia; wAIHA = warm antibody autoimmune hemolytic anemia; IMF = idiopathic myelofibrosis; ITP = immune thrombocytopenia.
